# Educational Attainment and Subjective Health and Well-Being; Diminished Returns of Lesbian, Gay, and Bisexual Individuals

**DOI:** 10.3390/bs9090090

**Published:** 2019-08-22

**Authors:** Shervin Assari, Mohsen Bazargan

**Affiliations:** 1 Department of Family Medicine, Charles R. Drew University of Medicine and Science, Los Angeles, CA 90059, USA; 2 Department of Family Medicine, UCLA, Los Angeles, CA 90095, USA

**Keywords:** lesbian, gay, and bisexual (LGB), sexual orientation, minorities, sexual minorities, socioeconomic position, socioeconomic status, poverty status, education, well-being, self-rated health

## Abstract

**Background:** Educational attainment is one of the strongest determinants of subjective health and well-being. Minorities’ Diminished Returns, however, suggests that such an effect may be smaller for the members of racial/ethnic minorities such as Blacks and Hispanics relative to non-Hispanic Whites. Only one study has previously shown that minorities’ diminished returns may also apply to lesbian, gay, and bisexual (LGB) individuals; however, that study has focused on other outcomes (i.e., obesity). **Aims:** To compare LGB and non-LGB American adults for the effects of educational attainment on subjective health and well-being. **Methods:** This cross-sectional study used baseline data of 31,480 adults in the Population Assessment of Tobacco and Health (PATH, 2013), a nationally representative study in the United States. The independent variable was educational attainment. The dependent variable was subjective health and well-being, measured using four items. Race, ethnicity, age, gender, poverty status, and employment were the covariates. LGB status was the moderator. **Results:** Overall, individuals with higher educational attainment had better subjective health and well-being. We found a significant interaction between LGB status and educational attainment which was suggestive of that the boosting effect of high educational attainment on better subjective health and well-being was systemically smaller for LGB than non-LGB individuals. **Conclusions:** In the United States, highly educated LGB adults experience poor subjective health and well-being, a status that is disproportionate to their educational attainment.

## 1. Background

According to Minorities’ Diminished Returns (MDRs) theory, which is supported by an extensive body of empirical evidence [1,2,3], at least some health disparities are due to less than expected protective effects of educational attainment on the health and well-being of minority groups compared to the majority group [1,2,3]. This theoretical framework proposes that: (a) health disparities are not all due to socioeconomic status (SES) disparities across social groups, but at least some of it is due to the MDRs (i.e., smaller health effects of SES indicators for the member of the minority groups compared to the general population), and (b) the health inequalities between the marginalized and non-marginalized groups widen, instead of narrowing, at higher educational levels. As a result, the solution to health disparities should go beyond reducing the inequalities in SES but also address stressors and barriers that cause poor health of highly educated members of minority groups [1,2,3].

Although MDRs are documented for subjective well-being and self-rated health [4,5,6], and members of minority groups remain at a higher risk of poor health despite their high educational attainment [1,2,3], this research has been mainly focused on comparison of racial and ethnic groups [1,2,3]. Only one study has shown MDRs for sexual minority populations [7]; however, that single study was on obesity [7], not self-rated health and well-being. In this study, which was published in 2019, lesbian, gay, and bisexual (LGB) individuals were at an increased risk of obesity across all levels of educational attainment. For LGB individuals, however, educational attainment may lend less protection against risk of obesity than non-LGB individuals [7]. The author argued that MDRs may be relevant to minority status, which was broadly defined. The author proposed any social marginalization and stigmatization may diminish the health gain that is expected to follow educational attainment [7]; however, more research is needed on the relevance of MDRs for other outcomes of LGB individuals.

### Aims

To better understand the underlying mechanisms behind health disparities in LGB individuals [8,9,10,11,12], we compared LGB and non-LGB adults with regard to the effect of educational attainment on subjective health and well-being in US adults. Built on a national sample of Americans, and informed by MDRs theory [13,14], we expected the protective effect of educational attainment on various domains of subjective well-being and health to be smaller for LGB people compare to non-LGB individuals. If MDRs are relevant to LGB people, we would expect poor subjective well-being and health in highly educated LGB people.

## 2. Methods

### 2.1. Design and Settings

This is a secondary analysis of Wave 1 of Population Assessment of Tobacco and Health (PATH) adult data. PATH has enrolled about 49,000 people of 12 years of age or older. Wave 1 data were collected in 2013–2014. Although PATH also has youth data, this study only includes adults. We used publicly available PATH data sets from the Inter-University Consortium for Political and Social Research, University of Michigan.

### 2.2. Sample and Sampling

The inclusion criteria in the PATH-Adult study was (1) civilian, (2) non-institutionalized, (3) United States population, and (4) 18 years of age or older. Sampling in the PATH study was a four-stage stratified area probability sample. First, a stratified sample of primary sampling units (PSUs) (*n* = 156) was selected. These PSUs were either a county or a group of counties. The second stage formed and sampled smaller geographical segments in each primary sampling unit. The third stage sampled residential addresses, using the US Postal Service Computerized Delivery Sequence Files. The fourth stage was selection of one person from each sampled household.

### 2.3. Analytical Sample

The current analysis is limited to adults who had valid data on sexual orientation and subjective health and well-being. Our final analytical sample was 31,480 adults.

### 2.4. Study Variables

All study variables were measured at an individual level. The dependent variable (outcome) was subjective health and well-being. The independent variable (predictor) was educational attainment. Race, ethnicity, age, gender, poverty status, and region were the covariates. Sexual orientation (LGB status) was the moderator.

*Race and Ethnicity.* Race was self-identified and operationalized as a dichotomous variable: Black = 1, White = 0. Ethnicity was also self-identified and operationalized as a dichotomous variable: Non-Hispanic = 0, Hispanic = 1.

*Demographic Covariates.* Demographic factors such as gender and age were considered. Gender was a dichotomous variable: male = 0, female = 1. Age was an interval measure, with a range from 1 to 7: (1) “18 to 24” years old, (2) “25 to 34” years old, (3) “35 to 44” years old, (4) “45 to 54” years old, (5) “55 to 64” years old, (6) “65 to 74” years old, and (7) “75+” years.

*SES Covariates.* SES covariates included employment and poverty status, which were both treated as dichotomous variables. Employment was coded as full-time employment versus any other conditions. Poverty status was coded as 0 for living below the 100% federal poverty line and 1 for living above the 100% federal poverty line.

*Independent Variables.* Educational attainment was made up of a six-level continuous variable as follows: (1) less than high school, (2) general educational development, (3) high school graduate, (4) some college (no degree) or associates degree, (5) bachelor’s degree, and (6) advanced degree.

*Outcome.* Our outcome was subjective health and well-being, measured using four items. Individuals reported their overall evaluation of (1) quality of life, (2) physical health, (3) mental health, and (4) overall health. Items were: (1) In general, how would you rate your physical health; excellent, very good, good, fair, or poor? (2) In general, how would you rate your mental health, which includes stress, depression, and problems with emotions? (3) In general, would you say your overall health is excellent, very good, good, fair, or poor? (4) In general, would you say your quality of life is excellent, very good, good, fair, or poor? As response options for all items were (1) poor, (2) fair, (3) good, (4) very good, or (5) excellent, varying 1 to 5, the total score had a range between 4 and 20, with a higher score indicating better subjective health and well-being.

*Moderator.* Sexual orientation, self-identified, was measured by asking the individuals about their sexual orientation. This variable was operationalized as non-LGB = 0 and LGB = 1.

### 2.5. Statistical Analysis

We analyzed the data using SPSS 23.0 (IBM Corporation, Armonk, NY, USA). We applied Taylor series linearization to re-estimate the variance of our variables using survey design variables such as weight, PSU, cluster, and strata. As a result of applying survey weights, the results are generalizable to the US general population. For univariate analysis, we reported frequencies and percentages as well as means and standard deviations. For multivariable analysis, we applied four linear regression models. First, we ran two linear regression models: *Model 1* only had the main effects of LGB status, educational attainment, and covariates. This model did not include any interaction terms. *Model 2* included the main effects of *Model 1* and also an interaction term between educational attainment and LGB status. We then ran two additional group-specific models: *Model 3* was performed for non-LGB people. *Model 4* was performed for LGB people. We reported unstandardized regression coefficient (b), standardized regression coefficient (B), standard error (SE), 95% CI, and *p* values.

### 2.6. Ethics

All the participants provided written informed consent. The Institutional Review Board of the Westat approved the PATH study protocol.

## 3. Results

### 3.1. Descriptive Statistics

This study included 31,480 American adults who were either non-LGB (*n* = 29,303, 93.1%) or LGB (*n* = 2177; 6.9%). Table 1 shows descriptive statistics of the overall sample as well as by sexual orientation (Table 1).

There were more Hispanics in the LGB than non-LGB group. There were more poor people in the LGB than non-LGB group. There were also more women in the LGB than non-LGB group. LGB and non-LGB group, however, did not differ in race. LGB people were younger than those in the non-LGB group. LGB people reported worse subjective health and well-being than non-LGB people (Table 1).

### 3.2. Multivariable Models in the Pooled Sample

Table 2 presents the summary of the results of two linear regression models with educational attainment as the independent variable and subjective health and well-being as the dependent variable. Both models were estimated in the overall sample. *Model 1* only entered the main effect of educational attainment and covariates. *Model 2*, however, also added an interaction term between sexual minority status and educational attainment.

Based on *Model 1*, high educational attainment was associated with better subjective health and well-being. *Model 2* showed a statistically significant interaction between LGB status and educational attainment on poor subjective health and well-being, suggesting that high educational attainment has diminished effect on the subjective health and well-being of LGB than non-LGB individuals (Table 2).

### 3.3. Multivariable Models in LGB and Non-LGB Individuals

Table 3 presents the results of two other linear regression models with educational attainment as the independent variable and better subjective health and well-being as the dependent variable. *Model 3* and *Model 4* were estimated for non-LGB and LGB people, respectively.

Based on *Model 3*, high educational attainment was associated with better subjective health and well-being in non-LGB individuals. *Model 4* also showed the same pattern for LGB individuals. The magnitude of the effect of educational attainment on subjective health and well-being was, however, larger for non-LGB (b = 0.58, *p* < 0.001) than LGB (b = 0.39, *p* < 0.001) individuals (Table 3).

## 4. Discussion

From our analysis it can be determined that high educational attainment was associated with better subjective health and well-being. LGB and non-LGB people, however, differed in such an effect. High educational attainment seemed to have a smaller boosting effect on subjective health and well-being of LGB than non-LGB individuals.

In line with other work that shows high educational attainment has smaller health effects on the health and well-being of racial and ethnic minority individuals than the general population [7,15,16,17], we showed poor subjective health and well-being in highly educated LGB individuals. Similar patterns are shown for a wide range of associations between SES indicators and health outcomes [13,14]; however, this literature has defined minority status based on race/ethnicity. The effects of education [4], income [18], employment [19], and marital status [20] on obesity [21], depression [22], anxiety [20], self-rated health [4], and chronic disease [23] are all smaller for racial and ethnic minorities than the majority group.

Only in a single recent study was educational attainment shown to have larger health effects for non-LGB people than LGB individuals. In that study, educational attainment showed stronger negative association with obesity in non-LGB than LGB people [7]. The current study should be regarded as the second study on MDRs of educational attainment for the LGB community and the first to show MDRs for subjective health and well-being of LGB people.

There is a need to understand the structural, psychological, and behavioral mechanisms that explain why educational attainment loses at least some of its protective effect for LGB individuals as well as some other minority individuals. One underlying mechanism may be stigmatization and marginalization that generate stress, discrimination, and poor access to new resources and poor chance to mobilize available resources [7]. For ethnic minority groups defined by race and ethnicity, labor market discrimination is shown to be a mechanism [4]. That is, educational attainment does not provide the same outcome of high economic utility in the daily life of individuals. So, highly educated people still remain marginalized and show risk behaviors [1,3], poor mental health [6], and physical health [21,24,25].

### 4.1. Implications

Elimination of health disparities is a strategic priority for the US government and local states. The results of the current study may suggest some new insight into how MDRs of marginalized groups may be one reason they remain at high risk of poor health despite their access to resources. We argue that the solution to health disparities between LGB and non-LGB people requires interventions which not only reduce SES gaps but also minimize MDRs of educational attainment for the members of marginalized groups.

There is a need for policies at a national and local level that address the existing inequalities and disparities in outcomes as well as those in social determinants. Such an approach would require the understanding that interventions on social determinants of health such as educational attainment may result in unbalanced impact for marginalized and non-marginalized groups, and thus may not reduce but increase the gap through MDRs of SES for marginalized people [2,3,4,5,14,18,20,21,26,27]. This notion suggests that universal interventions that increase educational attainment of all populations may in fact generate some disparities. We need policies and programs that disproportionately reach and help individuals that are members of marginalized groups [2,3,26]. Unfortunately, trust is lower in this marginalized population and we can hardly even reach those who are not publicly open about their sexual identity. Our argument here is that even for the section that we can reach, if we promote their SES indicators, their outcome would change by a lesser degree than expected. To eliminate health disparities in marginalized people such as LGB people, there is a need to address the barriers that minimize returns of available resources.

### 4.2. Limitations

This study had some methodological limitations. The cross-sectional design of our data does not allow for causal inferences. The sample size was imbalanced in non-LGB and LGB groups. This issue, however, only impacts our group specific model and not our pooled sample model with the interaction terms. Income, employment, marital status, and area level SES were missing. This study did not measure neighborhood characteristics or exposure to marketing and density of retails in the area. Finally, despite having controlled for covariates, LGB and non-LGB groups varied in gender/sex, educational attainment, and SES. These groups differences may have contributed to some of the differential effects of educational attainment in the LGB group. Despite these limitations, we believe this study does extend what is already known about health and well-being of sexual minority populations. The PATH study has a robust methodology that increases our trust in the results [28,29].

## 5. Conclusions

In the United States, the magnitude of the effect of educational attainment on subjective health and well-being is not similar between non-LGB than LGB individuals. Highly educated LGB people experience poor subjective health and well-being, which is independent of their SES. Health equity is not achievable in a society that treats its members differently. We should help all groups translate their resources to health equally.

## Figures and Tables

**Table 1:** Descriptive statistics.

	All		Non-LGB		LGB	
	n	%	n	%	n	%
LGB						
No	29,303	93.1	29,303	100.0	-	-
Yes	2177	6.9	-	-	2177	100.0
Race						
White	23,303	82.6	21,768	82.8	1535	80.8
Black	4892	17.4	4527	17.2	365	19.2
Ethnicity *						
Non-Hispanic	25,733	82.9	24,079	83.3	1654	77.3
Hispanic	5305	17.1	4820	16.7	485	22.7
Gender *						
Women	15,534	49.3	14,091	48.1	1443	66.3
Men	15,946	50.7	15,212	51.9	734	33.7
Poverty Status *						
Living in poverty	9739	33.9	8776	32.8	963	47.6
Living out of poverty	19,001	66.1	17,942	67.2	1059	52.4
Fulltime Employment *						
No	17,286	54.9	15,965	54.5	1321	60.7
Yes	14,194	45.1	13,338	45.5	856	39.3
Age (1–7) *	**Mean**	**SD**	**Mean**	**SD**	**Mean**	**SD**
	2.93	1.74	2.98	1.75	2.29	1.48
Educational Attainment (1–6)	3.53	1.37	3.54	1.37	3.39	1.40
Subjective Health and Well-Being *	11.09	3.51	11.17	3.49	10.09	3.56

* *p* < 0.05 for comparison of LGB and non-LGB individuals. Legend: LGB, lesbian, gay, and bisexual.

**Table 2:** Summary of linear regressions on subjective health and well-being in the pooled sample.

	b	SE	B	95% CI for b		*p*
**Model 1 (All)**						
Sexual orientation (LGB)	−1.01	0.08	−0.07	−1.18	−0.85	<0.001
Gender (male)	0.37	0.04	0.05	0.28	0.45	<0.001
Ethnicity (Hispanic)	0.06	0.06	0.01	−0.06	0.17	0.336
Race (Black)	0.25	0.06	0.03	0.14	0.36	<0.001
Age (years)	−0.34	0.01	−0.17	−0.36	−0.31	<0.001
Living out of poverty	0.96	0.05	0.13	0.86	1.06	<0.001
Fulltime employment	0.50	0.04	0.07	0.41	0.58	<0.001
Educational attainment (1–6)	0.56	0.02	0.22	0.53	0.60	<0.001
Constant	9.06	0.08		8.91	9.22	<0.001
**Model 2 (All)**						
Sexual orientation (LGB)	−0.49	0.22	−0.04	−0.92	−0.06	0.024
Gender (male)	0.37	0.04	0.05	0.29	0.45	<0.001
Ethnicity (Hispanic)	0.05	0.06	0.01	−0.06	0.17	0.359
Race (Black)	0.25	0.06	0.03	0.14	0.36	<0.001
Age (years)	−0.34	0.01	−0.17	−0.36	−0.31	<0.001
Living out of poverty	0.96	0.05	0.13	0.86	1.06	<0.001
Fulltime employment	0.50	0.04	0.07	0.41	0.58	<0.001
Educational attainment	0.57	0.02	0.22	0.54	0.61	<0.001
LGB × educational attainment	−0.15	0.06	−0.04	−0.27	−0.04	0.010
Constant	9.02	0.08		8.87	9.18	<0.001

Legend: b, unstandardized regression coefficient, B, unstandardized regression coefficient, SE, standard error.

**Table 3:** Summary of linear regression models on subjective health and well-being by sexual orientation.

	b	SE	B	95% CI for b		*p*
**Model 3 (Non-LGB)**						
Gender (male)	0.35	0.04	0.05	0.26	0.43	<0.001
Ethnicity (Hispanic)	0.01	0.06	0.00	−0.11	0.13	0.912
Race (Black)	0.23	0.06	0.03	0.12	0.35	<0.001
Age (years)	−0.35	0.01	−0.17	−0.37	−0.32	<0.001
Living out of poverty	0.95	0.05	0.13	0.84	1.05	<0.001
Fulltime employment	0.46	0.05	0.07	0.37	0.55	<0.001
Educational attainment (1–6)	0.58	0.02	0.23	0.54	0.61	<0.001
Constant	9.09	0.08		8.94	9.25	<0.001
**Model 4 (LGB)**						
Gender (male)	0.66	0.17	0.09	0.31	1.00	<0.001
Ethnicity (Hispanic)	0.47	0.21	0.05	0.06	0.87	0.026
Race (Black)	0.54	0.21	0.06	0.13	0.95	0.011
Age (years)	−0.22	0.06	−0.09	−0.34	−0.11	<0.001
Living out of poverty	1.05	0.19	0.15	0.69	1.42	<0.001
Fulltime employment	1.00	0.17	0.14	0.66	1.34	<0.001
Educational attainment (1–6)	0.39	0.06	0.15	0.26	0.52	<0.001
Constant	7.88	0.27		7.34	8.42	<0.001

b, unstandardized regression coefficient, B, unstandardized regression coefficient.
